# Cooperative Tracking of Vessel Trajectory by Multi-Static Passive Stations Using an MC-RMPF

**DOI:** 10.3390/s26113562

**Published:** 2026-06-03

**Authors:** Bingzhuo Liu, Lingqi Kong, Panlong Wu

**Affiliations:** 1 School of Automation, Nanjing University of Science and Technology, Nanjing 210094, China; lingqikong@njust.edu.cn; 2 Jiangsu Institute of Automation, Lianyungang 222061, China

**Keywords:** vessel trajectory tracking, station geometric distribution, multi-static passive radar stations, vessel motion constraints, resample–move particle filter

## Abstract

**Highlights:**

**What are the main findings?**

- Dynamic station subsets improve localization efficiency while keeping accuracy.
- RMPF with vessel-motion constraints enhances tracking accuracy and continuity.

**What are the implications of the main findings?**

- Geometry-aware station cooperation enables scalable passive maritime surveillance.
- Physics-informed particle filtering improves robust non-cooperative target tracking.

**Abstract:**

Traditional maritime vessel tracking methods based on multi-static passive radar stations typically process all available observations, leading to substantial computational overhead and estimation variance. Furthermore, discrepancies in refresh rates and noise levels among stations often cause significant jumps in estimated positions between updates, resulting in trajectory discontinuities. To mitigate these issues, this paper introduces a multi-station cooperative vessel tracking framework based on a motion-constrained resample–move particle filter (MC-RMPF). In the proposed method, systematic resampling is first used to alleviate particle degeneracy, and a markov chain monte carlo (MCMC) move step is subsequently applied to rejuvenate the resampled particles under vessel-motion feasibility constraints. Additionally, a distributed detection network is constructed using directional data from multiple stations, dynamically selecting optimal observation subsets to balance localization accuracy with computational load. The experimental results demonstrate that, compared to the baseline methods, our method reduces the Root Mean Square Error and Circular Error Probability of position tracking by 23.5% and 21.7%, respectively. It exhibits strong reliability in challenging scenarios such as target maneuvers and temporary observation loss.

## 1. Introduction

Maritime domain awareness is critical for a wide range of applications, including maritime traffic management, naval defense, illegal fishing monitoring, and search and rescue operations [1]. The cornerstone of this awareness is accurate and continuous vessel trajectory tracking. While the Automatic Identification System (AIS) is widely used, it is susceptible to intentional disabling and spoofing, or may simply be unavailable [2]. Therefore, non-cooperative tracking techniques that do not rely on vessels’ active transmission have become an indispensable complement.

The traditional radar systems utilized for this task are predominantly active, emitting electromagnetic waves and detecting the reflected signals. However, active radar systems suffer from several drawbacks: they are energy-intensive and susceptible to Anti-Radiation Missiles, and their emissions can be easily detected, compromising operational stealth [3]. In contrast, passive radar systems exploit existing illuminators of opportunity (e.g., FM radio, digital TV, and cellular signals) or, in the specific context of vessel tracking, the signals emitted by the vessels’ own radar systems [4]. This passive nature grants them significant advantages in terms of low cost, covert operation, and immunity to electronic countermeasures, making them an increasingly attractive solution for persistent surveillance [5].

However, passive trajectory tracking techniques still face many challenges. Radar measurements are highly susceptible to multipath propagation and atmospheric refraction in marine environments, leading to significant angular errors [6]. Vessel motion often exhibits irregular and dynamic trajectories that can hardly be captured by entirely statistically based filters [7]. Kinematic maneuvers like turns and speed changes lead to a progressive accumulation of vessel position errors [8]. Many existing studies employ predetermined, static configurations and neither optimize the station geometry nor leverage the potential of multi-station cooperation [9].

The fundamental principle of passive tracking relies on angle-of-arrival (AOA) [10], time-difference-of-arrival (TDOA) [11], or frequency-difference-of-arrival (FDOA) [12]. Although long-range maritime localization remains a key topic, short-range indoor tracking has recently gained attention, within which AOA-enabled millimeter-wave sensors have emerged due to their high positioning accuracy. However, noise introduced by low-cost hardware significantly degrades AOA estimation precision. To mitigate this issue, Ref. [10] proposed a data-driven deep learning regression model that effectively reduces AOA estimation errors. A similar challenge arises in medium- and long-range passive tracking. In this context, Ref. [13] addressed the impact of sensor position uncertainty on estimation performance and proposed a bias-compensated method to overcome the limitations of pseudo-linear estimators. Numerous studies have also focused on TDOA-based passive localization methods, in which the intersections of several hyperbolic surfaces correspond to the emitter’s position. Considering systems with more than three measurement nodes, Ref. [11] performed combinatorial analysis and compared a Cramér–Rao Lower Bound (CRLB)-based weighted averaging approach with the traditional Weighted Least Squares (WLS) method, demonstrating the superior positioning performance of the former. Nevertheless, this advantage diminishes when the number of TDOA nodes is limited.

Recent research has also explored hybrid AOA–TDOA measurements [14], in which the intersection of hyperbolic surfaces and bearing lines determines the source position. Since these intersections are inherently nonlinear and may yield multiple solutions, the Maximum Likelihood Estimation (MLE) is commonly employed. However, MLE performance strongly depends on the accuracy of the initial estimate during the tracking process. To alleviate this dependency, Ref. [15] introduced a Weighted Pseudo-Linear Estimator with Fusion (WPLE-F), which constructs an Instrumental Variable matrix to provide reliable initialization, thereby reducing sensitivity to initial conditions and achieving error performance close to the CRLB.

Early research mainly focused on bistatic or two-station configurations, which can resolve a target’s position through triangulation [16]. While simpler to implement, these systems are often limited by geometric dilution of precision (GDOP) and provide poor coverage and robustness, especially in complex coastal environments [17]. The concept of GDOP has been incorporated into inertial navigation and synthetic aperture radar systems to analyze the influence of external factors on navigation accuracy [18]. Mathematically, GDOP is derived from the inverse of the Fisher Information Matrix (FIM), and the D-optimality criterion optimizes the observation geometry—such as sensor placement—by maximizing the determinant of the FIM. Building upon this framework, Ref. [19] employed the FIM to propose a localization accuracy metric for optimal sensor deployment. Nevertheless, these approaches remain limited in long-range, non-line-of-sight scenarios that require multi-static cooperation.

The natural evolution is towards multi-station cooperative networks [20]. By fusing information from multi-static distributed passive radars, such networks can significantly improve localization accuracy, extend coverage, and enhance system reliability through redundancy [21]. The paradigm is shifting from simple data fusion to intelligent, cooperative tracking frameworks.

State estimation is the core component of a tracking system. The journey began with the Kalman Filter (KF) and its nonlinear extensions, such as the Extended KF (EKF) and Unscented KF (UKF), which are efficient for Gaussian linear or mildly nonlinear problems [22]. For more complex, highly nonlinear, and non-Gaussian scenarios encountered in real-world tracking, Sequential Monte Carlo methods, particularly the Particle Filter (PF), have gained prominence [23]. The PF represents the posterior probability density function by a set of random particles, offering remarkable flexibility.

However, standard PFs suffer from particle degeneracy and impoverishment [24]. A well-established remedy is the resample move strategy, in which importance resampling is followed by an MCMC move step to rejuvenate particles while preserving the target filtering distribution. This type of method combines importance sampling and MCMC sampling to mitigate progressive sample degeneration in dynamic Bayesian inference. It should be distinguished from MCMC-based particle filtering or sequential MCMC filtering methods, in which MCMC is used more fundamentally for sequential inference over the filtering distribution, often in high-dimensional multi-target tracking problems. For example, an MCMC-based particle filter was proposed for tracking a variable number of interacting targets, replacing the traditional importance sampling step with an MCMC sampling step [25]. Recently, a Rao–Blackwellised sequential MCMC sampler was developed for joint detection and tracking in clutter [26]. Despite these advances, significant challenges remain in the context of multi-station passive tracking:

- High computational cost: Most existing methods process all observation data from all stations. As the number of stations and targets grows, this “brute-force” fusion leads to prohibitive computational overhead, making it unsuitable for large-scale networks [27].
- Physical inconsistency: Many positioning algorithms merely rely on statistics and fail to incorporate domain knowledge such as velocity, tonnage inertia, and course heading. This often produces physically implausible trajectories with erratic jumps between updates, leading to discontinuous and unreliable tracks [28].

To bridge these critical gaps, this paper proposes a novel multi-station cooperative vessel tracking framework based on a physically constrained RMPF. Unlike traditional methods that treat data fusion and state estimation in isolation, our approach unifies geometric optimization with kinematic regularization to simultaneously enhance tracking efficiency and physical plausibility. The main contributions of this work are summarized as follows:

- A unified geometric modeling framework is established for multi-station AOA observations, in which the effects of station number and spatial configuration on positioning accuracy are rigorously analyzed within the GDOP framework, thereby providing a theoretical foundation for sensor layout optimization in passive tracking systems;
- A dynamic optimal subset selection strategy is introduced to obtain the most informative subset from the distributed stations, which can drastically reduce computational load while maintaining high-precision tracking.
- A physics-informed refinement mechanism is introduced, in which vessel kinematic constraints are embedded into the MCMC resampling procedure to suppress unrealistic state transitions and ensure physically consistent and dynamically reliable trajectory estimates.

The remainder of this paper is structured as follows. Section 2 formulates the AOA positioning model and introduces the dynamic optimal station subset selection strategy. Section 3 presents the proposed physics-constrained RMPF framework, detailing the integration of vessel kinetic constraints. Section 4 describes the implementation of this framework for trajectory tracking. Section 5 details the experimental setup and presents a comparative performance evaluation against baseline methods across multiple station configurations. Finally, Section 6 concludes the paper.

## 2. Preliminaries

### 2.1. Signal Discrimination

**Figure 1:**
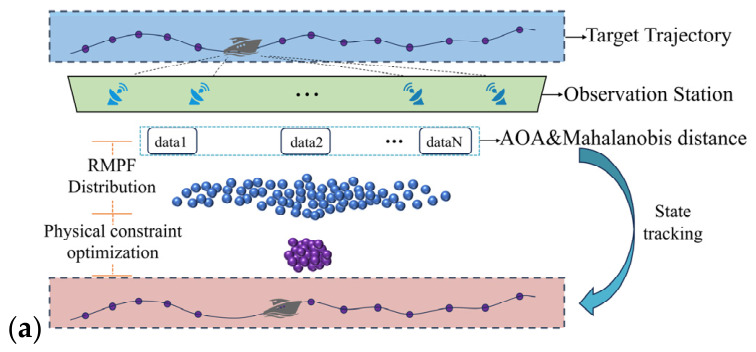

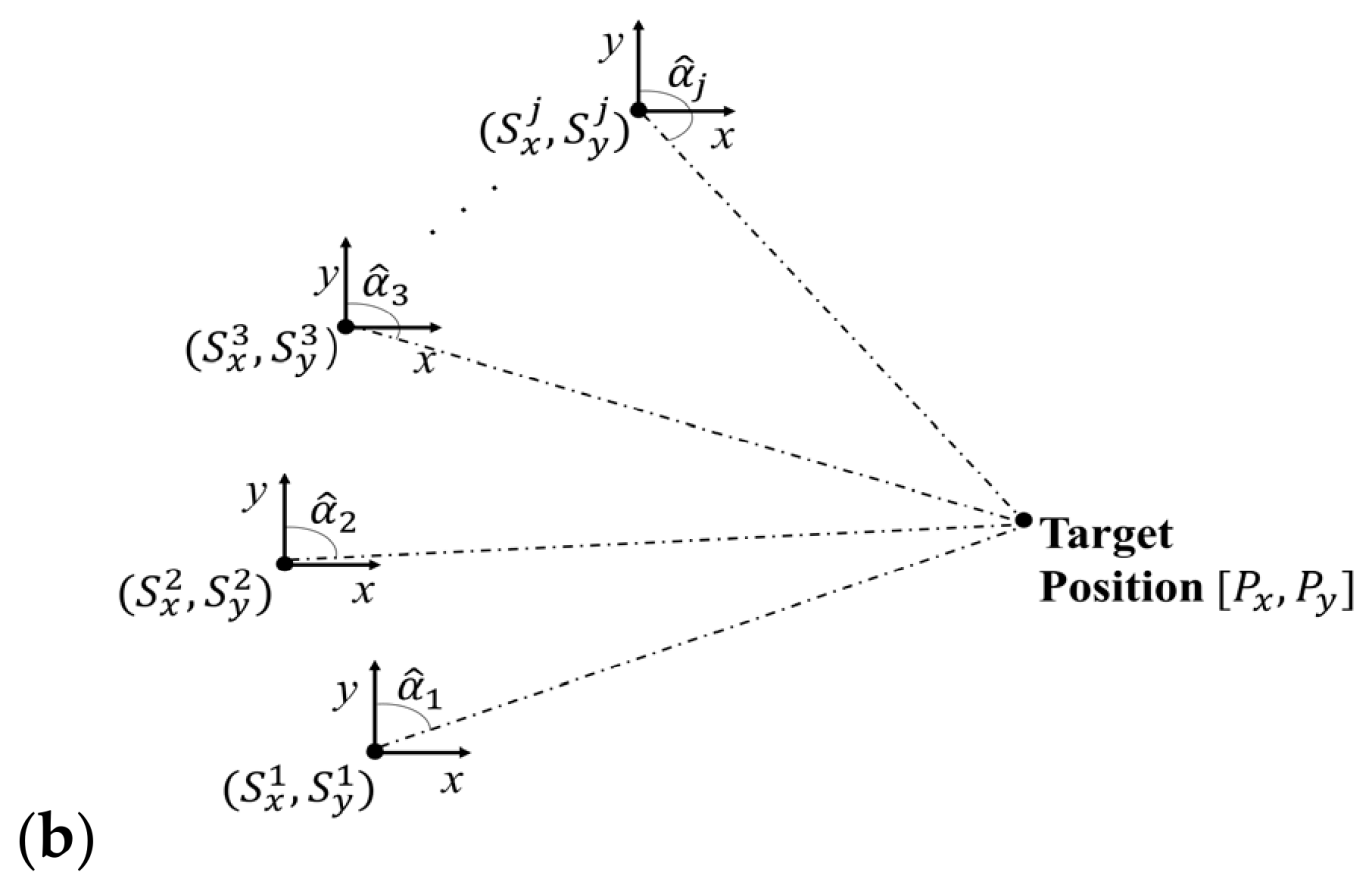
Signal model: (**a**) schematic diagram of ship trajectory target tracking, and (**b**) geometric configuration of multiple passive radar stations.

The surveillance network considered in this paper is a receive-only passive direction-finding system [5]. Each shore-based station intercepts vessel-emitted radio-frequency signals (e.g., navigation radar leakage or communication signals) and estimates the bearing of the source. No station transmits signals, and no reflected paths are utilized. Thus, the vessel acts as a non-cooperative emitter, and all stations operate as passive receivers, fundamentally differing from bistatic or multi-static radar systems. As illustrated in Figure 1, signal discrimination is the initial step in passive vessel tracking. Upon reception, raw AOA measurements are first calibrated to remove systematic biases. Subsequently, filtering and detection algorithms are applied to separate valid target signals from environmental noise.

Specifically, detection algorithms evaluate signal strength, frequency characteristics, and temporal stability; this is followed by feature extraction and classification to identify candidate target signals. Adaptive thresholding and time–frequency analysis further refine signal selection at each station. A probabilistic data association method based on Mahalanobis distance [29] is then employed for cross-station matching, effectively resolving ambiguities by incorporating station geometry, measurement accuracy, and historical trajectory information. Finally, triangulation is performed to estimate target positions after successful signal association. Detailed signal identification methods are beyond the scope of this paper.

### 2.2. Position Tracking Principle

As shown in Figure 1b, the multi-static passive system determines the position of a maritime target by fusing angular measurements from multiple stations, forming the basis of the proposed WLS model.

Consider a 2D scenario where the target is located at $\left(p_{x},p_{y}\right)$ and M passive stations are positioned at $\left(s_{x}^{j},s_{y}^{j}\right)$, j=1,2,…,M. Under ideal conditions, the bearing angle $\alpha_{j}$ measured by the jth station satisfies [13]

(1) $$sin\alpha_{j}(p_{x}-s_{x}^{j})-cos\alpha_{j}(p_{y}-s_{y}^{j})=0$$

In practice, angular measurements are subject to systematic and random errors, causing the measured value ${\hat{\alpha }}_{j}$ to deviate from the true value $\alpha_{j}$ by:

(2) $$\begin{array}{c} {\hat{\alpha }}_{j}=\alpha_{j}+\Delta \alpha_{j} \end{array}$$

where $\Delta \alpha_{j}∼N(0,\sigma_{j}^{2})$. Rearranging (1) yields

(3) $$\begin{array}{c} s_{x}^{j}tan\alpha_{j}-s_{y}^{j}=p_{x}tan\alpha_{j}-p_{y} \end{array}$$

Substituting ${\hat{\alpha }}_{j}$ and introducing noise $\eta_{j}$, we obtain

(4) $$\begin{array}{c} s_{x}^{j}tan{\hat{\alpha }}_{j}-s_{y}^{j}=p_{x}tan{\hat{\alpha }}_{j}-p_{y}+\eta_{j} \end{array}$$

where $\eta_{j}$ is the equivalent linearized measurement error induced by the angular error $\Delta \alpha_{j}$. Its variance is not identical to $\sigma_{\alpha j}^{2}$, but is obtained through first-order error propagation and depends on the target station geometry. For all M stations, the following linear system can be established:

(5) $$\begin{array}{c} z=Hp+\eta \end{array}$$

where

(6a) $$z=\left[\begin{array}{c} s_{x}^{1}sin{\hat{\alpha }}_{1}-{cos{\hat{\alpha }}_{1}s}_{y}^{1}\\ s_{x}^{2}sin{\hat{\alpha }}_{2}-{cos{\hat{\alpha }}_{2}s}_{y}^{2}\\ \vdots \\ s_{x}^{M}sin{\hat{\alpha }}_{M}-{cos{\hat{\alpha }}_{M}s}_{y}^{M} \end{array}\right],H=\left[\begin{array}{cc} sin{\hat{\alpha }}_{1} & -cos{\hat{\alpha }}_{1}\\ sin{\hat{\alpha }}_{2} & -cos{\hat{\alpha }}_{2}\\ \vdots & \vdots \\ sin{\hat{\alpha }}_{M} & -cos{\hat{\alpha }}_{M} \end{array}\right],$$

(6b) $$p=\left[\begin{array}{c} p_{x}\\ p_{y} \end{array}\right],\eta =\left[\begin{array}{c} \eta_{1}\\ \eta_{2}\\ \vdots \\ \eta_{M} \end{array}\right].$$

To account for the differing measurement accuracies across stations, a WLS scheme is adopted by constructing a diagonal weight matrix W as:

(7) $$W=diag(w_{1},\ldots ,w_{M})$$

where $w_{j}=1/\sigma_{j}^{2}$ is inversely proportional to the angular measurement accuracy of the corresponding station. The WLS estimate [15] of the target position is then

(8) $$\begin{array}{c} {\hat{p}}_{WLS}=(H^{T}WH{)}^{-1}H^{T}Wz \end{array}$$

A unique solution exists when M≥2 and the stations are not collinear, ensuring $H^{T}WH$ is non-singular.

### 2.3. Dynamic Selection of Optimal Station Subset

Although station locations are fixed, target–station geometry varies with vessel motion. A subset with good angular diversity at one epoch may become ill-conditioned elsewhere; thus, fixed offline selection is suboptimal. The proposed method dynamically updates the active subset based on the predicted target position to maintain favorable geometry.

GDOP is fundamentally defined as [30]:

(9) $$\mathrm{GDOP}=\sqrt{\sigma_{p_{x}}^{2}+\sigma_{p_{y}}^{2}}$$

where $\sigma_{p_{x}}$ and $\sigma_{p_{y}}$ are the standard deviations of positioning errors, respectively. From the AOA model, error propagation is obtained by differentiating Equation (1):

$$\begin{array}{cc} d\alpha_{j} & =\frac{\partial \alpha_{j}}{\partial p_{x}}dp_{x}+\frac{\partial \alpha_{j}}{\partial p_{y}}dp_{y}+\frac{\partial \alpha_{j}}{\partial s_{x}^{j}}ds_{x}^{j}+\frac{\partial \alpha_{j}}{\partial s_{y}^{j}}ds_{y}^{j}\\ & =\frac{\cos^{2}\alpha_{j}}{p_{x}-s_{x}^{j}}dp_{y}-\frac{\sin\alpha_{j}\cos\alpha_{j}}{p_{x}-s_{x}^{j}}dp_{x} \end{array}$$

(10) $$-\frac{{cos}^{2}\alpha_{j}}{p_{x}-s_{x}^{j}}ds_{y}^{j}+\frac{sin\alpha_{j}cos\alpha_{j}}{p_{x}-s_{x}^{j}}ds_{x}^{j}.$$

Then, the error propagation mechanism can be arranged as:

(11) dα=G⋅dp+ΔS

where $d\alpha =[d\alpha_{1},d\alpha_{2},\ldots ,d\alpha_{M}{]}^{T}$ is the angle deviation vector, dp is the position error vector, G is the observation matrix the j-th row of which is $\left[-\frac{sin\alpha_{j}cos\alpha_{j}}{p_{x}-s_{x}^{j}},\frac{{cos}^{2}\alpha_{j}}{p_{x}-s_{x}^{j}}\right]$, and ΔS means additional terms arising from station position uncertainties.

Given the WLS weight matrix W, the position error covariance matrix can be derived as:

(12) $$C_{p}=(G^{T}WG{)}^{-1}G^{T}W\Sigma_{\alpha }^{\mathrm{eq}}WG(G^{T}WG{)}^{-1}$$

where $\Sigma_{\alpha }^{\mathrm{eq}}$ is the equivalent angle-domain covariance matrix that incorporates both angular measurement noise and station position uncertainty.

To ensure consistent units, station position errors are projected into the angle domain. From Equation (10), the partial derivatives of the AOA with respect to station coordinates are

(13) $$\frac{\partial \alpha_{j}}{\partial s_{x}^{j}}=\frac{p_{y}-s_{y}^{j}}{r_{j}^{2}},\;\;\frac{\partial \alpha_{j}}{\partial s_{y}^{j}}=-\frac{p_{x}-s_{x}^{j}}{r_{j}^{2}}$$

where $r_{j}=\sqrt{(p_{x}-s_{x}^{j}{)}^{2}+(p_{y}-s_{y}^{j}{)}^{2}}$ is the target-to-station distance. Let $J_{s,j}=\left[\frac{\partial \alpha_{j}}{\partial s_{x}^{j}},\frac{\partial \alpha_{j}}{\partial s_{y}^{j}}\right]$ denote the corresponding Jacobian with respect to the j-th station’s position. Assuming the station position error is isotropic with covariance $\Sigma_{s,j}=\sigma_{s_{j}}^{2}I_{2}$,

(14) $$\sigma_{\alpha ,s_{j}}^{2}=J_{s,j}\Sigma_{s,j}J_{s,j}^{T}={\left(\frac{p_{y}-s_{y}^{j}}{r_{j}^{2}}\right)}^{2}\sigma_{s_{j}}^{2}+{\left(-\frac{p_{x}-s_{x}^{j}}{r_{j}^{2}}\right)}^{2}\sigma_{s_{j}}^{2}=\frac{\sigma_{s_{j}}^{2}}{r_{j}^{2}}$$

Notably, $\sigma_{\alpha ,s_{j}}^{2}$ has units of $m^{2}/m^{2}={\mathrm{rad}}^{2}$ (under the small-angle approximation), which is dimensionally compatible with the angular measurement variance $\sigma_{\alpha_{j}}^{2}$ (also in ${\mathrm{rad}}^{2}$). Therefore, the total equivalent angular variance for the j-th station is the sum of these two independent contributions:

(15) $$\sigma_{\alpha_{j},\mathrm{eq}}^{2}=\sigma_{\alpha_{j}}^{2}+\frac{\sigma_{s_{j}}^{2}}{r_{j}^{2}}$$

Consequently, the equivalent angle covariance matrix can be written as:

(16) $$\Sigma_{\alpha }^{\mathrm{eq}}=diag{(\sigma }_{\alpha_{1},\mathrm{eq}}^{2},\sigma_{\alpha_{2},\mathrm{eq}}^{2},\ldots ,\sigma_{\alpha_{M},\mathrm{eq}}^{2})$$

Ultimately, the GDOP for 2D positioning is obtained from the diagonal elements of $C_{p}$:

(17) $$\mathrm{GDOP}=\sqrt{\sigma_{p_{x}}^{2}+\sigma_{p_{y}}^{2}}=\sqrt{C_{p}\left(1,1\right)+C_{p}\left(2,2\right)}=\sqrt{\mathrm{trace}\left(C_{p}\right)}$$

If only angular noise is considered, the GDOP can be simplified as:

(18) $$\mathrm{GDOP}=\sqrt{\mathrm{trace}((H^{T}WH{)}^{-1})}.$$

For a system consisting of n observation stations, the optimal subset $S^{*}$ of size k is

(19) $$S^{*}=arg\underset{\begin{array}{c} S\subset 1,2,\ldots ,n\\ ∥S∥=k \end{array}}{\min}{GDOP}_{S}.$$

Exhaustive search is prohibitive; thus, an incremental greedy strategy is adopted. Under approximate submodularity, let $f(S)=-{GDOP}_{S}$ denote the utility function. For any subsets A⊂B and an element e∉B, the utility gain is assumed to satisfy the relaxed inequality:

(20) f(A∪e)−f(A)≥f(B∪e)−f(B)−ϵ

where ϵ≥0 is a relaxation parameter representing the deviation from strict submodularity. The algorithm proceeds as follows:

(i) initialize $S_{0}$ via exhaustive tri-station search;
(ii) iteratively add the station maximizing GDOP reduction;
(iii) terminate when |S|=k or improvement <ε

Since the GDOP reduction approximately exhibits a diminishing return behavior, the greedy strategy is used as a computationally efficient heuristic rather than as a strictly optimal solver. The relaxation parameter ϵ characterizes the deviation from exact submodularity; therefore, the greedy solution may deviate from the ideal approximation bound by a residual term that increases with ϵ. In this study, the greedy result is further verified by numerical GDOP comparison.

To quantify the contribution of an individual station to positioning accuracy, a Station Impact Factor (*SIF*) is defined:

(21) $${SIF}_{i}=\frac{{GDOP}_{S\setminus i}-{GDOP}_{S}}{{GDOP}_{S}}\times 100\%.$$

For any station i∈S, its *SIF* value satisfies

(22) $${SIF}_{i}\approx \frac{\lambda_{i}}{\sum_{j\in S}\lambda_{j}}\times 100\%$$

where $\lambda_{i}$ is the contribution of station i to the Fisher information matrix. Therefore, *SIF* reflects the importance of a certain station to the overall geometric configuration.

To address practical complexities, an adaptive weighting mechanism is proposed as:

(23) $$w_{i}=\alpha \cdot w_{rel,i}+\beta \cdot w_{acc,i}+\gamma \cdot w_{geo,i}$$

where $w_{rel,i}$, $w_{acc,i}$, and $w_{geo,i}$ represent station reliability, measurement accuracy, and geometric contribution, respectively, and α, β, γ are weight coefficients satisfying α+β+γ=1. Then, the modified GDOP calculation formula is

(24) $${GDOP}_{w}=\sqrt{tr((G^{T}W^{-1}G{)}^{-1})}$$

where $W=diag(w_{1}^{-1},w_{2}^{-1},...,w_{n}^{-1})$ is the weight matrix.

For a system with n observation stations, complexity analysis shows that: (1) the initial selection phase requires evaluating GDOP values for all three-station combinations, with complexity $O((\frac{n}{3}))$; (2) the greedy iteration phase involves at most (k−3) iterations, each computing GDOP for (n−|S|) candidates, yielding $O((k-3)(n-\frac{k+2}{2}))$; and (3) the dynamic update phase recalculates GDOP for k stations after substitution, with complexity O(k(n−k)).

## 3. Improved RMPF Considering Physical Constraints Regarding Vessel Motion

### 3.1. PF and Systematic Resampling

In PF [23], the posterior density is approximated by weighted particles $\{x_{k}^{i},w_{k}^{i}{\}}_{i=1}^{N}$, where N is the number of sampling particles, $x_{k}^{i}$ is the state sample and $w_{k}^{i}$ its weight:

(25) $$p\left(x_{k}|z_{1:k}\right)\approx \sum_{i=1}^{N}{\overset{∼}{w}}_{k}^{i}\delta \left(x_{k}-x_{k}^{i}\right)$$

where $\sum_{i=1}^{N}{\overset{∼}{w}}_{k}^{i}=1$, normalized as ${\overset{∼}{w}}_{k}^{i}$; δ(⋅) is the *Dirac* function. The particles are typically drawn from a selected importance density function $q(x_{k}|x_{k-1}^{i},z_{k})$, with weights:

(26) $$w_{k}^{i}\propto w_{k-1}^{i}\frac{p\left(z_{k}|x_{k}^{i}\right)p\left(x_{k}^{i}|x_{k-1}^{i}\right)}{q\left(x_{k}^{i}|x_{k-1}^{i},z_{k}\right)}.$$

PFs suffer from particle degeneracy, in which weight variance increases over time. This is mitigated using systematic resampling, triggered by the effective sample size [24].

(27) $$N_{\mathrm{eff},k}=\frac{1}{\sum_{i=1}^{N}({\overset{∼}{w}}_{k}^{\left(i\right)}{)}^{2}}$$

where ${\overset{∼}{w}}_{k}^{\left(i\right)}$ is the normalized particle weight, and the range of $N_{eff,k}$ is [1,N]. When $N_{eff,k}<N_{th}$, the resampling is triggered. Compared with multinomial resampling, systematic resampling reduces variance and has O(N) complexity. To adapt resampling frequency, a dynamic threshold is used:

(28) $$N_{th}\left(k\right)=\alpha \cdot N_{th}\left(k-1\right)+\left(1-\alpha \right)\cdot \beta \cdot N$$

where α is a smoothing factor and β controls the baseline ratio.

The PF described above recursively approximates the filtering posterior $p(x_{k}|z_{1:k})$ by the weighted empirical measure $\{x_{k}^{\left(i\right)},w_{k}^{\left(i\right)}{\}}_{i=1}^{N}$, with the resampled set asymptotically distributed according to $p\left(x_{k}|z_{1:k}\right)$. Although resampling alleviates weight degeneracy, it induces particle impoverishment; the resampled set contains many duplicates and fails to explore the support of $p(x_{k}|z_{1:k})$ adequately.

### 3.2. Metropolis–Hastings Move

To rejuvenate resampled particles while preserving the target filtering distribution, the MC-RMPF employs a Metropolis–Hastings (MH) move. Comprehensive treatments of MCMC theory can be found in Ref. [31].

Let π(x) denote the local target distribution that the MH chain is designed to leave invariant. Given a proposal density $q(x^{\prime }|x)$, the MH acceptance probability is

(29) $$\begin{array}{c} \alpha (x\to x^{\prime })=min\left\{1,\frac{\pi (x^{\prime })q(x|x^{\prime })}{\pi (x)q(x^{\prime }|x)}\right\} \end{array}$$

and the associated transition kernel admits the standard form:

(30) $$\begin{array}{c} K(x,B)=\int_{B}q(x^{\prime }|x)\alpha (x\to x^{\prime })dx^{\prime }+r(x)\delta_{x}(B) \end{array}$$

where $r(x)=1-\int q(x^{\prime }|x)\alpha (x\to x^{\prime })dx^{\prime }$ is the rejection mass.

Two clarifying remarks are in order. First, the first-order Markov property invoked by MCMC concerns the algorithmic state visited by the chain, not the underlying physical variable $X_{n}$. Any finite-memory dependence in the vessel-dynamics model can be recast as a first-order Markov chain by augmenting the algorithmic state; therefore, the Markov assumption here imposes no modeling restriction on vessel motion. Second, memorylessness is necessary but not sufficient for convergence to π. The kernel must additionally satisfy the π-invariance condition:

(31) $$\begin{array}{c} \int \pi (x)K(x,B)dx=\pi (B) \end{array}$$

together with mild ergodicity conditions (irreducibility and aperiodicity). The MH construction in Equations (29) and (30) satisfies the detailed balance equation:

(32) $$\begin{array}{c} \pi (x)q(x^{\prime }|x)\alpha (x\to x^{\prime })=\pi (x^{\prime })q(x|x^{\prime })\alpha (x^{\prime }\to x) \end{array}$$

which implies π-invariance, while ergodicity follows from standard regularity conditions on the proposal.

### 3.3. Integration of RMPF with Physical Constraints

#### 3.3.1. Target Distribution of the Constrained MCMC Kernel

Before deriving the acceptance ratio, we explicitly specify the stationary distribution that the proposed MCMC kernel is designed to leave invariant. This clarification is essential because, in our framework, the MCMC step does not target the unconstrained filtering posterior $p(x_{k}|z_{1:k})$, but rather a constraint-modified conditional posterior defined as follows.

For each resampled particle indexed by ancestor $a_{k}^{\left(i\right)}$, the MCMC chain at time k is constructed to be invariant with respect to

(33) $$\begin{array}{c} \pi_{k}\left(x_{k}|x_{k-1}^{a_{k}^{\left(i\right)}},z_{1:k}\right)\propto p(z_{k}|x_{k})p\left(x_{k}|x_{k-1}^{a_{k}^{\left(i\right)}}\right)\phi \left(x_{k},x_{k-1}^{a_{k}^{\left(i\right)}}\right) \end{array}$$

where ϕ(⋅,⋅)∈[0,1] is the physical feasibility factor encoding vessel kinematic constraints, to be specified in Equation (36) below. Three remarks clarify the role of $\pi_{k}$:

(i) Conditioning on the ancestor. The target $\pi_{k}$ is conditional on the specific ancestor particle $x_{k-1}^{a_{k}^{\left(i\right)}}$ associated with the resampled particle being moved. This conditioning is intrinsic to the resample–move framework and ensures that the MCMC step refines the local conditional distribution rather than the global marginal.
(ii) Constraint modification. The factor ϕ distinguishes $\pi_{k}$ from the standard filtering posterior. Specifically, $\pi_{k}$ is the constraint-restricted posterior: it concentrates probability mass on the kinematically feasible region of state space while preserving Bayesian consistency with the observation $z_{k}$.
(iii) Relationship to the filtering posterior. Marginalizing $\pi_{k}$ over ancestor indices with weights $\left\{w_{k}^{\left(i\right)}\right\}$ recovers the constraint-modified filtering posterior:

(34) $$\begin{array}{c} \overset{∼}{p}(x_{k}|z_{1:k})\propto p(x_{k}|z_{1:k})\bar{\phi }(x_{k}) \end{array}$$

where $\bar{\phi }(x_{k})=\sum_{i}W_{k}^{\left(i\right)}\phi \left(x_{k},x_{k-1}^{\left(a_{k}^{\left(i\right)}\right)}\right)$ is the marginalized feasibility factor. Consequently, the proposed algorithm targets $\overset{∼}{p}$ rather than $p(x_{k}|z_{1:k})$—a deliberate design choice motivated by the unphysical behavior of unconstrained PF outputs documented in Section 2.

#### 3.3.2. Constraint-Modified Acceptance Ratio

To sample from $\pi_{k}$, a constrained proposal distribution is constructed as:

(35) $$\begin{array}{c} x_{k}^{\prime }=q\left(x_{k}^{\prime }|x_{k}^{\left(i\right)}\right) \end{array}$$

where $x_{k}^{\prime }$ denotes the candidate state and q(⋅∣⋅) is the proposal density.

In this paper we adopt a soft-constraint specification:

(36) $$\begin{array}{c} \phi (x_{k},x_{k-1})=exp(-\lambda \Phi (x_{k},x_{k-1})) \end{array}$$

where $\Phi (x_{k},x_{k-1})\geq 0$ is a weighted sum of normalized violations in speed v, turning radius r, and acceleration a:

(37) $$\Phi \left(x_{k},x_{k-1}\right)=w_{v}[v/v_{max}-1{]}_{+}+w_{r}[1-r/r_{min}{]}_{+}+w_{a}[\left|a\right|/a_{max}-1{]}_{+}$$

with $\left[\cdot {]}_{+}=max(\cdot ,0\right)$ and λ>0 controlling constraint strictness. Unlike a hard indicator 𝟙$1_{F}$, the soft specification assigns low but nonzero probability to near-infeasible states, preserving filter adaptability under extreme maritime conditions while maintaining physical consistency.

The parameters in the motion feasibility factor are not treated as universal constants. Instead, they are specified according to the vessel class, navigation scenario, and available prior information. In practical deployment, the limits $v_{max}$, $a_{max}$, and $r_{min}$ can be obtained from vessel type-dependent maneuvering characteristics and historical AIS statistics. The penalty weights $w_{v}$, $w_{a}$, and $w_{r}$ determine the relative importance of speed, acceleration, and turning-radius violations, respectively, while λ controls the overall softness of the constraint. In this study, these parameters were fixed before testing and were selected using independent validation trajectories rather than the evaluation trajectories. Therefore, the proposed soft constraint should be understood as a scenario dependent feasibility model rather than a universal vessel motion law.

Given the target $\pi_{k}$ in Equation (33), the Metropolis–Hastings acceptance ratio is

(38) $$\begin{array}{c} \alpha \left(x_{k}^{\left(i\right)}\to x_{k}^{\prime }\right)=min\left\{1,\frac{\pi_{k}(x_{k}^{\prime }|x_{k-1}^{\left(a_{k}^{\left(i\right)}\right)},z_{1:k})q(x_{k}^{\left(i\right)}|x_{k}^{\prime })}{\pi_{k}(x_{k}^{\left(i\right)}|x_{k-1}^{\left(a_{k}^{\left(i\right)}\right)},z_{1:k})q(x_{k}^{\prime }|x_{k}^{\left(i\right)})}\right\} \end{array}$$

Substituting Equation (34) into the above expression gives

(39) $$\begin{array}{c} \alpha \left(x_{k}^{\left(i\right)}\to x_{k}^{\prime }\right)=min\left\{1,\frac{p(z_{k}|x_{k}^{\prime })p(x_{k}^{\prime }|x_{k-1}^{\left(a_{k}^{\left(i\right)}\right)})\phi (x_{k}^{\prime },x_{k-1}^{\left(a_{k}^{\left(i\right)}\right)})q(x_{k}^{\left(i\right)}|x_{k}^{\prime })}{p(z_{k}|x_{k}^{\left(i\right)})p(x_{k}^{\left(i\right)}|x_{k-1}^{\left(a_{k}^{\left(i\right)}\right)})\phi (x_{k}^{\left(i\right)},x_{k-1}^{\left(a_{k}^{\left(i\right)}\right)})q(x_{k}^{\prime }|x_{k}^{\left(i\right)})}\right\} \end{array}$$

For the symmetric Gaussian random-walk proposal used in this work, namely,

(40) $$q\left(x_{k}^{\prime }|x_{k}^{\left(i\right)}\right)=q\left(x_{k}^{\left(i\right)}|x_{k}^{\prime }\right)$$

the proposal–density ratio cancels, and the acceptance probability reduces to

(41) $$\alpha \left(x_{k}^{\left(i\right)}\to x_{k}^{\prime }\right)=min\left\{1,\frac{p(z_{k}|x_{k}^{\prime })p(x_{k}^{\prime }|x_{k-1}^{\left(a_{k}^{\left(i\right)}\right)})\phi (x_{k}^{\prime },x_{k-1}^{\left(a_{k}^{\left(i\right)}\right)})}{p(z_{k}|x_{k}^{\left(i\right)})p(x_{k}^{\left(i\right)}|x_{k-1}^{\left(a_{k}^{\left(i\right)}\right)})\phi (x_{k}^{\left(i\right)},x_{k-1}^{\left(a_{k}^{\left(i\right)}\right)})}\right\}$$

Thus, kinematically infeasible candidates are not rejected by an additional constrained proposal. Instead, they receive a lower acceptance probability through the feasibility ratio. This avoids the introduction of an intractable proposal normalizing constant and preserves a clear Metropolis–Hastings construction with respect to the constraint-modified target distribution. At each MCMC step, the candidate $x_{k}^{\prime }$ is accepted with probability α; otherwise, the current particle $x_{k}^{\left(i\right)}$ is retained.

#### 3.3.3. Invariance and Convergence

The MH transition kernel K defined by the proposal q and the acceptance probability α satisfies detailed balance with respect to $\pi_{k}$:

(42) $$\begin{array}{c} \pi_{k}(x_{k})q(x_{k^{\prime }}|x_{k})\alpha (x_{k}\to x_{k^{\prime }})=\pi_{k}(x_{k^{\prime }})q(x_{k}|x_{k^{\prime }})\alpha (x_{k^{\prime }}\to x_{k}) \end{array}$$

which is verified by direct substitution of Equations (33), (35) and (37). Detailed balance implies $\pi_{k}$-invariance, and irreducibility and aperiodicity follow from the strictly positive support of the Gaussian base proposal q, combined with ϕ>0 almost everywhere on the feasible region. Therefore, the constrained MCMC kernel converges to the constraint-modified conditional posterior $\pi_{k}$, and the overall algorithm converges to the constraint-modified filtering posterior $\overset{∼}{p}$ defined in Equation (34).

This integration yields a physically informed Bayesian framework that preserves probabilistic consistency while incorporating motion constraints, improving robustness and preventing implausible state transitions.

## 4. Implementing the MC-RMPF for Vessel Trajectory Tracking

**Figure 2:**
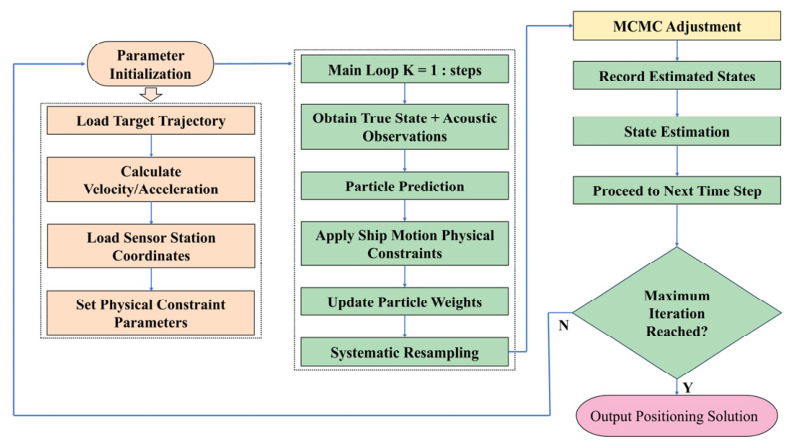
Flowchart of the constructed MC-RMPF tracking framework.

The implementation of the physically constrained MC-RMPF for vessel tracking is illustrated in Figure 2. By integrating MCMC sampling with motion constraints, the method mitigates particle degeneracy and impoverishment, improving robustness and accuracy. The recursive procedure is as follows.

### 4.1. Initialization

If the prior distribution of the vessel target’s state at time k=0 is obtained as $p(x_{0})$, sample N particles from it:

(43) $$x_{0}^{\left(i\right)}∼p\left(x_{0}\right),i=1,2,\ldots ,N$$

Initial weights are uniformly assigned as:

(44) $$w_{0}^{\left(i\right)}=\frac{1}{N},i=1,2,\ldots ,N$$

Subsequently, the filtering process is executed recursively through the following “prediction—update” steps.

### 4.2. Prediction Step and Update Step

- Prediction Step (Time Update)

Given the posterior particle set $\{x_{k-1}^{\left(i\right)},w_{k-1}^{\left(i\right)}{\}}_{i=1}^{N}$ at time k−1, the filtering proceeds recursively.

(45) $$x_{k}^{i}=f_{k-1}\left(x_{k-1}^{\left(i\right)}\right)+v_{k-1}^{\left(i\right)}$$

where i=1,2,…,N, $f_{k-1}(\cdot )$ is the system state transition function; $v_{k-1}^{\left(i\right)}$ is the process noise, typically assumed to be Gaussian white noise $v_{k-1}^{\left(i\right)}∼N(0,Q_{k-1})$. The weights remain unchanged in this step, resulting in the predicted particle set $\left\{x_{k}^{\left(i\right)},w_{k-1}^{\left(i\right)}\right\}$.

- Update Step (Measurement Update)

Given measurement $z_{k}$, the likelihood is

(46) $$p\left(z_{k}|x_{k}^{\left(i\right)}\right)=\frac{1}{\sqrt{\left(2\pi {)}^{n_{z}}|R_{k}\right|}}exp\left(-\frac{1}{2}[z_{k}-h_{k}(x_{k}^{\left(i\right)}){]}^{T}R_{k}^{-1}[z_{k}-h_{k}(x_{k}^{\left(i\right)})]\right)$$

where $h_{k}(\cdot )$ denotes the measurement equation, $R_{k}$ is the measurement noise covariance matrix, and $n_{z}$ is the dimension of the measurement. Then, update the weights:

(47) $$w_{k}^{i}=w_{k-1}^{i}\cdot p\left(z_{k}|x_{k}^{i}\right),i=1,2,\ldots ,N$$

Normalize the weights:

(48) $$w_{k}^{i}=\frac{w_{k}^{i}}{\sum_{j=1}^{N}w_{k}^{j}},i=1,2,\ldots ,N$$

Thus, the posterior approximation $\left\{x_{k}^{i},w_{k}^{i}\right\}$ is obtained.

### 4.3. Degeneracy Detection and Systematic Resampling

After weight normalization, particle degeneracy may occur, where a few particles dominate. This is quantified by the effective sample size:

(49) $$N_{eff,k}=\frac{1}{\sum_{i=1}^{N}({\overset{∼}{w}}_{k}^{\left(i\right)}{)}^{2}}.$$

When $N_{eff,k}<N_{threshold}$, the following systematic resampling procedure is applied to alleviate particle degeneracy and restore diversity.

The cumulative weight distribution is constructed as:

(50) $$c_{0}=0,c_{i}=c_{i-1}+w_{k}^{i},i=1,2,\ldots ,N$$

Next, a uniformly distributed random number $u_{1}∼U[0,1/N]$ is generated to determine the initial position of the resampling grid. For each particle index j=1,2,…,N, calculate $u_{j}=u_{1}+\left(j-1\right)/N$, find i such that $c_{i-1}<u_{j}\leq c_{i}$, set the resampled particle as $x_{k}^{\left(j\right)}=x_{k}^{\left(i\right)}$, and reset its weight to $w_{k}^{\left(j\right)}=1/N$.

This procedure replicates high-weight particles and removes low-weight ones, reducing variance but potentially causing sample impoverishment.

### 4.4. Physically Constrained MCMC Move Step

To restore diversity and enforce physical feasibility, a constrained MCMC move is applied after resampling. Each particle $x_{k}^{\left(i\right)}$ initializes a short Markov chain, and candidates are drawn from the constrained proposal $q(x_{k}^{\prime }|x_{k}^{\left(i\right)})$. Acceptance follows the Metropolis–Hastings rule in Equation (37), preserving detailed balance.

During implementation, the MCMC procedure proceeds as follows:

- Initialization

For each resampled particle i=1,2,…,N, set the initial state $x_{k}^{\left(i,0\right)}=x_{k}^{\left(i\right)}$. Specify the burn-in period B and the number of iterations M.

- Candidate sampling and acceptance

At each iteration m=1,2,…,M+B, generate a proposal $x_{k}^{*}∼N\left(x_{k}^{\left(i,m-1\right)},\Sigma \right)$, and evaluate its acceptance probability according to Equation (37). The candidate state is accepted with probability α, otherwise the previous state is retained.

- State update

Upon completion of the burn-in period, the final sample $x_{k}^{\left(i,m\right)}$ is retained as the updated particle for time step k. The covariance Σ of the proposal distribution can be adaptively adjusted based on the empirical covariance of the particle set. Subsequently, the filtered state $x_{k|k}$ and covariance $P_{k|k}$ are estimated as:

(51) $$x_{k|k}=\sum_{i=1}^{N}w_{k}^{i}x_{k}^{i}$$

(52) $$P_{k|k}=\sum_{i=1}^{N}w_{k}^{i}(x_{k}^{i}-x_{k|k})(x_{k}^{i}-x_{k|k}{)}^{T}.$$

To enhance adaptability and convergence stability, the proposal covariance and resampling threshold are dynamically adjusted by

(53) $$\Sigma_{k}=\beta \cdot P_{k|k}$$

(54) $$N_{threshold}=\gamma \cdot N,0<\gamma <1$$

where β is a scaling coefficient typically in [0.1, 2], and γ determines the sensitivity of the resampling trigger.

## 5. Experiments and Analysis

### 5.1. Experimental Scenario

**Figure 3:**
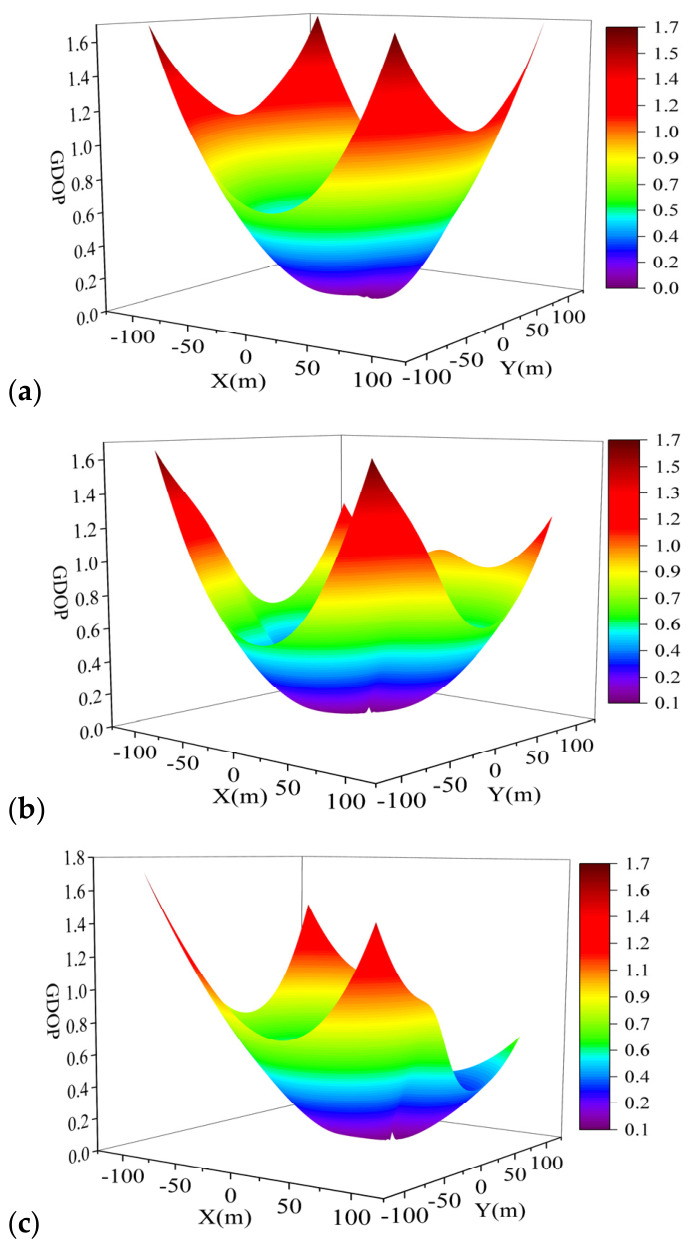
GDOP distribution heat maps for arrangements of (**a**) rectangular, (**b**) triangular, and (**c**) L-shaped arrays of stations.

GDOP evaluates the impact of observation station geometry on positioning accuracy. Table 1 and Figure 3 present the GDOP distributions for typical layouts. The L-shaped configuration achieves the minimal average GDOP (0.261) and the maximum effective coverage (GDOP < 0.3), outperforming the rectangular (average = 0.283) and triangular (average = 0.271) arrays. Consequently, the L-shaped layout provides a more uniform precision distribution and is adopted for subsequent experiments.

### 5.2. Experimental Configurations

The experiments are conducted within a 500 km × 500 km sea region utilizing dual-station, tri-station, and multi-station (7 stations) configurations, as detailed in Table 2. The modified MC-RMPF tracking algorithm is implemented with N=1000 particles. Tracking sessions last 120 min with a synchronized 1 Hz sampling frequency. The directional measurements contain Gaussian noise with an angular standard deviation of $0.15^{\circ }$ (an accuracy of $\pm 0.15^{\circ }$ at 2σ), while the environmental process noise standard deviation is estimated at 50 m.

The same noise statistics are also used for the theoretical lower-bound analysis. Specifically, the angular measurement covariance matrix $R_{k}$ is constructed from the station-dependent AOA noise variance, while the process noise covariance matrix $Q_{k}$ is determined according to the environmental process noise used in the simulations. Therefore, the CRLB/PCRLB calculation and the Monte Carlo tracking experiments are based on consistent probabilistic assumptions.

**Figure 4:**
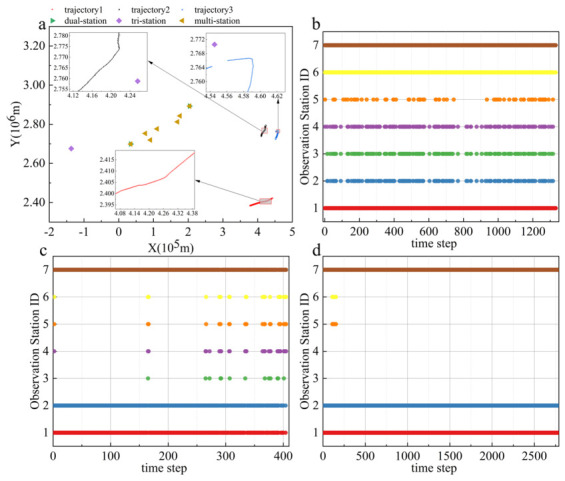
Target maneuver trajectories and dynamic observation station subset selection. (**a**) 2D plot of target maneuver trajectories (1–3). The insets provide detailed views of the local trajectory segments utilizing dual-, tri-, and multi-station configurations. (**b**–**d**) Temporal distribution of the selected dynamic observation station subsets corresponding to Trajectory 1, Trajectory 2, and Trajectory 3, respectively.

Figure 4a illustrates three representative maneuvering ship trajectories comprising both linear segments and coordinated turns. The first trajectory spans 6593 s, extending from 121°49′31″ E, 21°33′5″ N (UTM: 378,360.25 m, 2,383,631.45 m) to 122°26′47″ E, 21°53′50″ N (UTM: 442,814.06 m, 2,421,554.01 m). The second trajectory lasts 5361 s, ranging from 122°7′2″ E, 24°46′14″ N (UTM: 410,752.12 m, 2,739,830.16 m) to 122°14′10″ E, 25°15′31″ N (UTM: 423,076.86 m, 2,793,802.70 m). The third trajectory covers 8802 s, proceeding from 122°32′3″ E, 24°58′45″ N (UTM: 452,984.63 m, 2,762,721.61 m) to 122°31′26″ E, 24°38′1″ N (UTM: 451,813.90 m, 2,724,462.47 m).

### 5.3. Algorithm Performance Comparison Analysis

This study evaluates the proposed kinematically constrained MC-RMPF against standard PF, APF, MCMC-PF [23], and IMM-UKF. To demonstrate the dynamic station selection strategy, Figure 4a presents the three maneuvering trajectories, while Figure 4b–d illustrate the temporal evolution of the active observation subsets. Determined by the Incremental Greedy Algorithm (Section 2), this dynamic mechanism adaptively reconfigures the sensor network based on target–sensor geometry, thereby optimizing GDOP and reducing redundant computational burden.

**Figure 5:**
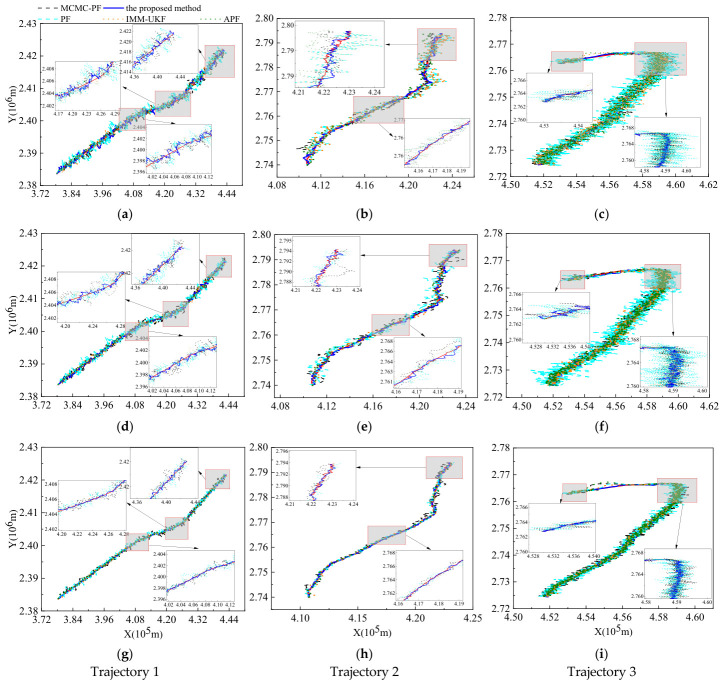
Comparison of trajectory estimation results for maneuvering targets using PF, MCMC-PF, IMM-UKF, APF, and the proposed method. The results are categorized by observation station configuration: (**a**–**c**) Dual-station configuration for Trajectories 1, 2, and 3, respectively; (**d**–**f**) Tri-station configuration for Trajectories 1, 2, and 3, respectively; and (**g**–**i**) Multi-station configuration for Trajectories 1, 2, and 3, respectively. The zoomed-in insets illustrate local tracking details, highlighting the stability of each algorithm during complex maneuvers.

Figure 5 provides a comprehensive tracking evaluation across dual-station (Figure 5a–c), tri-station (Figure 5d–f), and multi-station (Figure 5g–i) configurations. As expected, increasing the number of observation stations improves geometric diversity and reduces estimation uncertainty across all methods. However, performance disparities remain substantial. Under the dual-station setup, standard PF and MCMC-PF exhibit pronounced trajectory jitter and occasional divergence during turning maneuvers. In contrast, the proposed method (solid blue curve) consistently achieves the closest alignment with the reference state across all configurations. The magnified insets confirm that incorporating vessel motion kinetics effectively constrains state evolution to physically plausible regions, suppressing spurious fluctuations during high-dynamic maneuvers.

To quantitatively evaluate tracking performance, six statistical metrics are employed: Average Displacement Error (ADE), Final Displacement Error (FDE), Average Root Mean Square Error (ARMSE), Standard Deviation of RMSE (SDRMSE), Symmetric Mean Absolute Percentage Error (SMAPE), and Circular Error Probable (CEP50). Let $x_{k}$ and ${\hat{x}}_{k}$ denote the reference state and estimated positions at time step k, respectively. The metrics are defined as [28]:

(55) $$ADE=\frac{1}{N}\sum_{k=1}^{N}\sqrt{(x_{k}-{\hat{x}}_{k}{)}^{2}+(y_{k}-{\hat{y}}_{k}{)}^{2}}$$

(56) $$FDE=\sqrt{(x_{N}-{\hat{x}}_{k}{)}^{2}+(y_{N}-{\hat{y}}_{k}{)}^{2}}$$

(57) $$ARMSE=\frac{1}{N}\sum_{k=1}^{N}{RMSE}_{k}$$

(58) $${RMSE}_{k}=\sqrt{\frac{1}{M}\sum_{m=1}^{M}\left[(x_{k}^{m}-{\hat{x}}_{k}^{m}{)}^{2}+(y_{k}^{m}-{\hat{y}}_{k}^{m}{)}^{2}\right]}$$

(59) $$SDRMSE=\sqrt{\frac{1}{N}\sum_{k=1}^{N}({RMSE}_{k}-ARMSE{)}^{2}}$$

(60) $$SMAPE=\frac{100\%}{N}\sum_{k=1}^{N}\frac{\sqrt{(x_{k}-{\hat{x}}_{k}{)}^{2}+(y_{k}-{\hat{y}}_{k}{)}^{2}}}{\frac{1}{2}\left(\sqrt{{\hat{x}}_{k}^{2}+{\hat{y}}_{k}^{2}}+\sqrt{x_{k}^{2}+y_{k}^{2}}\right)}$$

(61) $$CEP50=Median\left({\left\{\sqrt{(x_{k}-{\hat{x}}_{k}{)}^{2}+(y_{k}-{\hat{y}}_{k}{)}^{2}}\right\}}_{k=1}^{N}\right)$$

To further evaluate the statistical efficiency of the proposed method, a posterior Cramér–Rao Lower Bound (PCRLB) is introduced as a theoretical benchmark. Since the vessel tracking problem is nonlinear, the bound is computed using the locally linearized state-transition and AOA measurement models. Let $F_{k}$ denote the Jacobian of the state transition function, $H_{k}$ denote the Jacobian of the AOA measurement function with respect to the target state, and $Q_{k}$ and $R_{k}$ denote the process noise and measurement noise covariance matrices, respectively. The recursive information form approximation of the PCRLB is given by [11]

(62) $$P_{k|k-1}^{\mathrm{LB}}=F_{k}P_{k-1}^{\mathrm{LB}}F_{k}^{T}+Q_{k}$$

(63) $$P_{k}^{\mathrm{LB}}={\left[{\left(P_{k|k-1}^{\mathrm{LB}}\right)}^{-1}+H_{k}^{T}R_{k}^{-1}H_{k}\right]}^{-1}$$

The position domain lower bound is then computed as:

(64) $${\mathrm{PCRLB}}_{p,k}=\sqrt{\mathrm{tr}\left(SP_{k}^{\mathrm{LB}}S^{T}\right)}$$

where S is the selection matrix that extracts the two-dimensional position components from the state vector. In addition, the empirical position error covariance of each algorithm is calculated from repeated Monte Carlo runs:

(65) $$C_{e,k}^{\left(m\right)}=\frac{1}{N_{\mathrm{MC}}-1}\sum_{r=1}^{N}\left(e_{k,r}^{\left(m\right)}-{\bar{e}}_{k}^{\left(m\right)}\right){\left(e_{k,r}^{\left(m\right)}-{\bar{e}}_{k}^{\left(m\right)}\right)}^{T}$$

(66) $$\sigma_{p,k}^{2,(m)}=\mathrm{tr}\left(C_{e,k}^{\left(m\right)}\right)$$

where $e_{k,r}^{\left(m\right)}={\hat{p}}_{k,r}^{\left(m\right)}-p_{k}$ is the position estimation error of method m in the r-th Monte Carlo run. This allows the proposed method to be compared not only with other algorithms, but also with the theoretical lower bound under the same noise assumptions.

**Figure 6:**
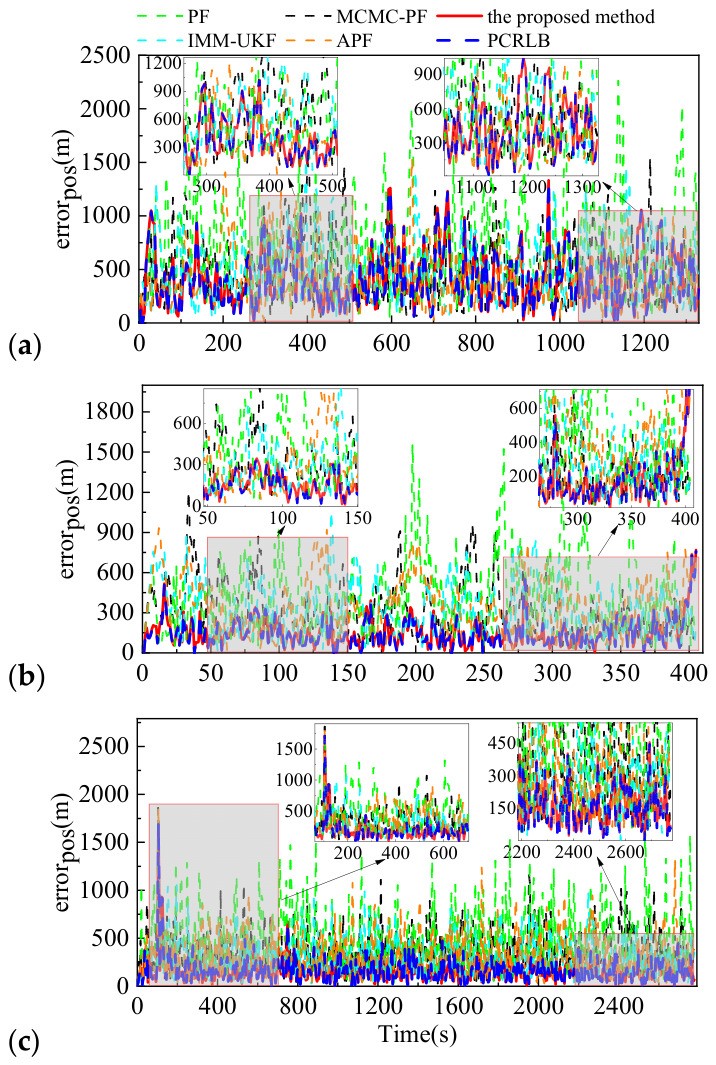
Temporal evolution of position estimation errors for Trajectories 1–3 under the dual-station configuration. The performance of the proposed method is compared against PF, MCMC-PF, IMM-UKF, APF algorithms and PCRLB. (**a**) Trajectory 1. (**b**) Trajectory 2. (**c**) Trajectory 3.

**Figure 7:**
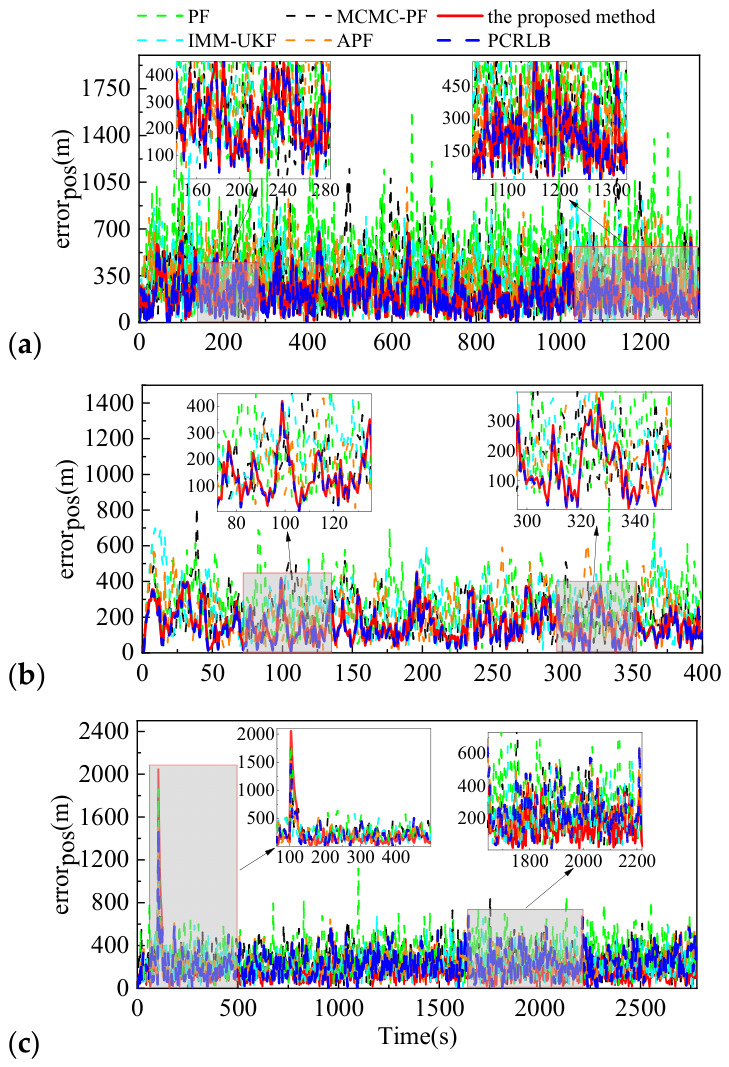
Temporal evolution of position estimation errors for Trajectories 1–3 under the tri-station configuration. (**a**) Trajectory 1. (**b**) Trajectory 2. (**c**) Trajectory 3.

**Figure 8:**
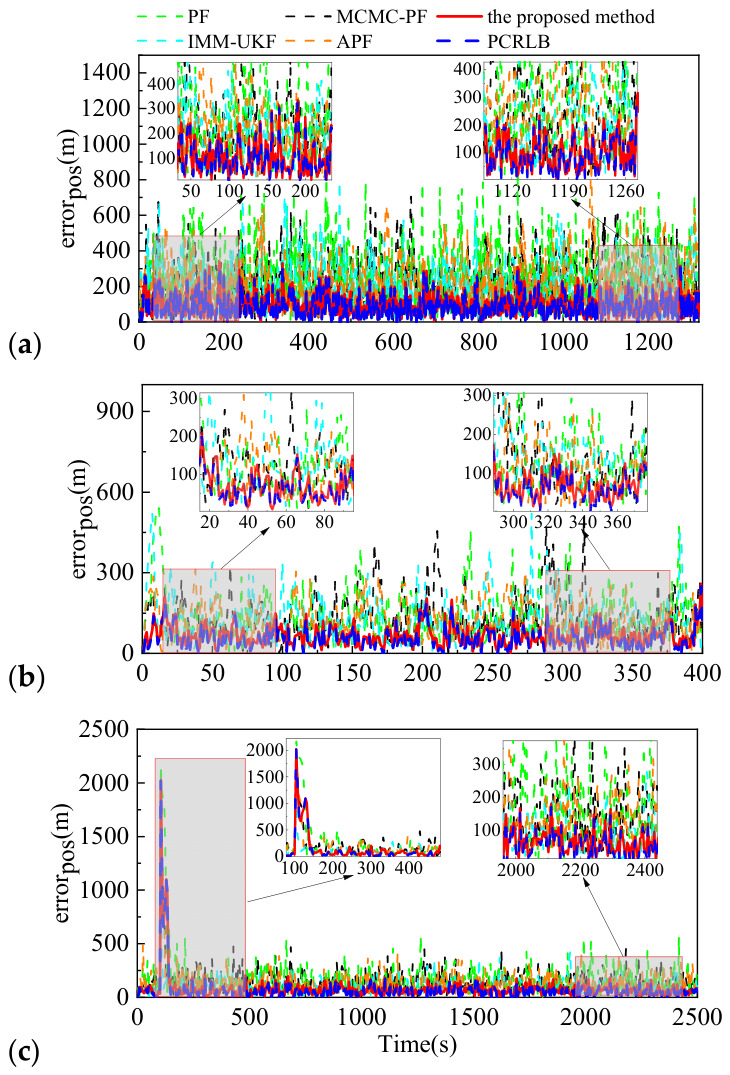
Temporal evolution of position estimation errors for Trajectories 1–3 under the multi-station configuration. (**a**) Trajectory 1. (**b**) Trajectory 2. (**c**) Trajectory 3. The inset in (**c**) provides a detailed view of the error dynamics during a peak fluctuation event.

Table 3, Table 4 and Table 5 and Figure 6, Figure 7 and Figure 8 summarize the statistical metrics and temporal position errors. In the dual-station scenario (Figure 6), limited geometric diversity induces severe error spikes for standard PF, reaching an ARMSE of 1809.99 m and an SDRMSE of 990.99 m (Table 3). As configurations expand, error magnitudes decrease monotonically. The proposed method’s ARMSE is reduced from 594.23 m (dual-station) to 483.30 m (multi-station). Under the multi-station scenario (Table 5), the proposed framework consistently achieves the lowest global errors, attaining an ADE of 402.45 m and a CEP50 of 339.38 m, significantly outperforming MCMC-PF (ADE: 646.87 m) and IMM-UKF (ADE: 610.37 m). Furthermore, the magnified inset in Figure 8c demonstrates that the proposed method ensures faster reconvergence and tighter error bounds under transient disturbances. Table 6 reports execution time and floating-point operations measured on an AMD Ryzen 7 processor. While the proposed method incurs higher computational overhead due to the MCMC move step and dynamic subset selection, the additional cost remains tractable and yields substantial gains in estimation accuracy and robustness.

Table 7 compares the empirical position-error variance of different algorithms with the PCRLB under different station configurations. The empirical variance is calculated from the dispersion of the position-estimation errors, while the PCRLB is obtained using the same process-noise and AOA measurement-noise statistics as those used in the simulation. It can be observed that all empirical variances are above the PCRLB, which is consistent with the theoretical lower-bound property. Among the compared methods, the proposed MC-RMPF achieves the smallest empirical variance in all station configurations and is closest to the PCRLB. This indicates that the proposed motion-constrained resample–move mechanism not only reduces the mean tracking error but also suppresses error dispersion during maneuvering target tracking.

### 5.4. Measurement Error Sensitivity Analysis

**Figure 9:**
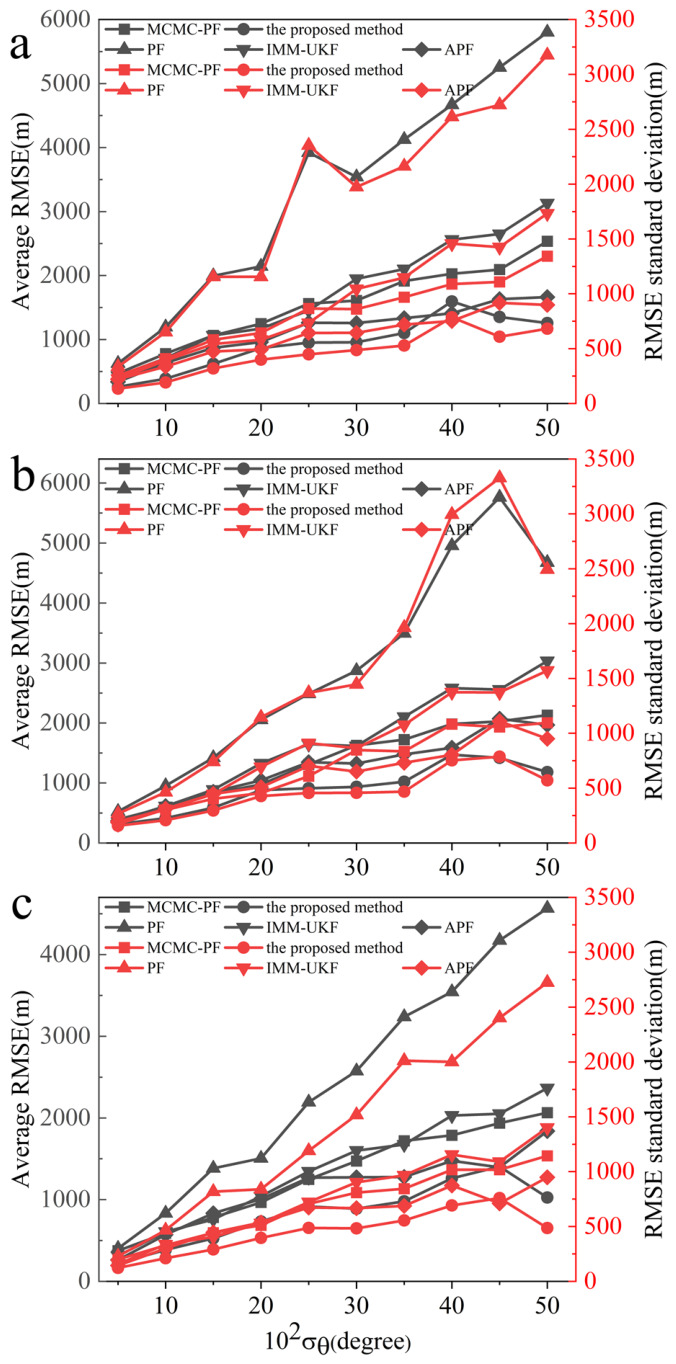
Sensitivity analysis of tracking performance with respect to angular measurement noise. The Average RMSE (left axis) and RMSE Standard Deviation (right axis) are plotted against noise levels ranging from 0.05° to 0.5°. (**a**) Dual-station configuration. (**b**) Tri-station configuration. (**c**) Multi-station configuration.

To evaluate robustness against measurement uncertainty, angular measurement noise was varied from 0.05° to 0.5°. As depicted in Figure 9, estimation errors for all algorithms exhibit an approximately linear growth with increasing noise levels. However, distinct performance tiers are evident. While PF and IMM-UKF demonstrate significant noise sensitivity—especially under the dual-station configuration Figure 9a—the proposed method consistently maintains the lowest Average RMSE and RMSE Standard Deviation across all geometric layouts, ensuring superior accuracy and stability under uncertain conditions.

**Figure 10:**
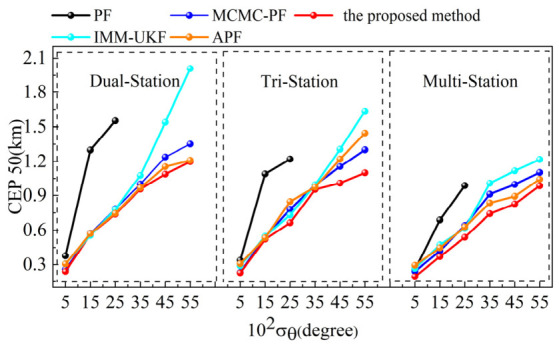
Comparison of the Circular Error Probable (CEP50) versus angular measurement noise across different station configurations. The panels display the performance of the five filtering algorithms under (**Left**) dual-station, (**Middle**) tri-station, and (**Right**) multi-station layouts.

Figure 10 further corroborates these findings using the CEP50 metric. The proposed algorithm achieves the smallest CEP50 radius across the entire noise spectrum. Notably, beyond a specific divergence threshold around 0.3°, the performance gap between the proposed framework and conventional baselines widens significantly. Unlike standard stochastic methods that struggle with increased variance, the integration of physical motion constraints effectively suppresses unrealistic state transitions, preventing the filter from diverging even when severe measurement noise induces resampling saturation.

## 6. Conclusions

This study proposes a physically constrained MC-RMPF framework for vessel tracking, integrating geometric optimization, dynamic station selection, and kinematic constraints. Comprehensive evaluations across dual-, tri-, and multi-station scenarios demonstrate its superior accuracy and adaptability compared to baseline algorithms. Specifically, embedding physical motion priors effectively suppresses trajectory divergence during high-dynamic maneuvers, while the L-shaped layout with an incremental greedy strategy optimally balances precision and computational overhead. Quantitative metrics, including GDOP, RMSE, and CEP50, confirm consistent performance under realistic marine conditions and remarkable resilience against escalating angular measurement noise. These results underscore the advantage of embedding domain-aware physical priors into Bayesian filtering to enhance state estimation reliability in complex maritime environments.

## Figures and Tables

**Table 1:** Geometric arrangement of different types of observation stations.

Arrangement Type	Position Coordinates (m)	Minimum GDOP	Average GDOP
Rectangular Arrangement	(−20, 10), (20, 10) (−20, −10), (20, −10)	0.096	0.283
Triangular Arrangement	(0, 30), (−25.98, −15) (25.98, −15)	0.091	0.271
L-shaped Arrangement	(0, 0), (50, 0), (0, 50) (25, 25)	0.064	0.261

**Table 2:** Observation and station configurations.

Observation Configuration		Latitude and Longitude	UTM Projection Coordinates
Dual-station	station 1	118°24′56″, 24°20′06″	34,596, 2,699,000 m
	station 2	120°02′30″, 26°07′26″	204,167, 2,892,770 m
Tri-station	station 1	116°44′45″, 24°03′52″	−136,640, 2,675,570 m
	station2	118°30′13″, 27°13′59″	54,545.7, 3,020,260 m
	station 3	120°42′03″, 28°58′39″	275,981, 3,207,670 m
Multi-station	station 1	118°24′56″, 24°20′06″	34,596, 2,699,000 m
	station 2	118°57′54″, 24°32′07″	91,088.2, 2,719,480 m
	station 3	118°48′40″, 24°50′05″	76,490.9, 2,753,160 m
	station 4	119°08′27″, 25°03′14″	110,552, 2,776,470 m
	station 5	119°42′43″, 25°23′30″	169,147, 2,812,390 m
	station 6	119°46′41″, 25°40′09″	176,550, 2,842,990 m
	station 7	120°02′30″, 26°07′26″	204,167, 2,892,770 m

**Table 3:** Statistical performance metrics of trajectory estimation in the dual-station scenario.

Method	ADE	FDE	ARMSE	SDRMSE	SMAPE	CEP50
PF	1514.85	1931.67	1809.99	990.99	0.1353%	1309.21
MCMC-PF	943.89	369.98	1086.32	537.94	0.0837%	873.98
IMM-UKF	952.97	1039.66	1143.66	632.54	0.0846%	794.85
APF	661.69	515.87	775.17	403.96	0.0598%	593.73
The proposed method	498.95	329.63	594.23	322.87	0.0437%	429.25

**Table 4:** Statistical performance metrics of trajectory estimation in the tri -station scenario.

Method	ADE	FDE	ARMSE	SDRMSE	SMAPE	CEP50
PF	1390.34	722.97	1187.75	1237.39	0.1048%	1067.64
MCMC-PF	737.47	649.23	861.47	445.44	0.0657%	668.92
IMM-UKF	727.70	502.93	847.77	435.11	0.0639%	730.33
APF	650.68	431.38	733.28	337.89	0.0673%	647.82
The proposed method	479.75	798.66	557.66	284.42	0.0423%	433.79

**Table 5:** Statistical performance metrics of trajectory estimation in the multi-station scenario.

Method	ADE	FDE	ARMSE	SDRMSE	SMAPE	CEP50
PF	1161.20	908.85	1408.52	797.51	0.1024%	977.55
MCMC-PF	646.87	548.31	755.55	390.54	0.0584%	575.33
IMM-UKF	610.37	335.50	719.04	380.23	0.0551%	539.33
APF	604.25	784.55	631.65	350.03	0.0639%	434.06
The proposed method	402.45	836.20	483.30	267.71	0.0360%	339.38

**Table 6:** Computational complexity of different methods across various scenarios.

Method		PF	MCMC-PF	IMM-UKF	APF	Proposed
Dual-Station	Time (s)	0.443	2.420	0.453	0.489	4.512
	Flops	3.45 × 10^7^	6.97 × 10^7^	1.84 × 10^6^	3.93 × 10^7^	6.97 × 10^7^
Tri-Station	Time (s)	0.499	2.732	0.466	0.548	4.667
	Flops	4.05 × 10^7^	8.99 × 10^7^	2.34 × 10^6^	5.55 × 10^7^	8.99 × 10^7^
Multi-Station	Time (s)	0.607	2.859	0.572	0.619	4.935
	Flops	5.99 × 10^7^	1.10 × 10^8^	2.23 × 10^6^	7.17 × 10^7^	1.10 × 10^8^

**Table 7:** Average empirical position error variance and PCRLB under different station configurations. Unit: (m^2^).

Configuration	PF Var.	MCMC-PF Var.	IMM-UKF Var.	APF Var.	Proposed Var.	Mean PCRLB
Dual-Station	$9.82\;\times \;10^{5}$	$2.89\times 10^{5}$	$4.00\times 10^{5}$	$1.63\times 10^{5}$	$1.04\times 10^{5}$	$9.84\times 10^{4}$
Tri-Station	$1.53\times 10^{6}$	$1.98\times 10^{5}$	$1.89\times 10^{5}$	$1.14\times 10^{5}$	$8.09\times 10^{4}$	$7.92\times 10^{4}$
Multi-Station	$6.36\times 10^{5}$	$1.53\times 10^{5}$	$1.45\times 10^{5}$	$1.23\times 10^{5}$	$7.17\times 10^{4}$	$6.50\times 10^{4}$

## Data Availability

The relevant data of this study are available on request from the corresponding author (P. W.) via the email address: plwu@njust.edu.cn.
